# A bacteriophage cocktail targeting *Yersinia pestis* provides strong post-exposure protection in a rat pneumonic plague model

**DOI:** 10.1128/spectrum.00942-24

**Published:** 2024-09-18

**Authors:** Paul B. Kilgore, Jian Sha, Emily K. Hendrix, Blake H. Neil, William S. Lawrence, Jennifer E. Peel, Lauren Hittle, Joelle Woolston, Alexander Sulakvelidze, Jennifer A. Schwartz, Ashok K. Chopra

**Affiliations:** 1 Department of Microbiology and Immunology, University of Texas Medical Branch, Galveston, Texas, USA; 2 Institute for Human Infections & Immunity, and the Galveston National Laboratory, University of Texas Medical Branch, Galveston, Texas, USA; 3 Intralytix, Inc., Columbia, Maryland, USA; 4 Sealy Institute for Vaccine Sciences, University of Texas Medical Branch, Galveston, Texas, USA; 5 Center for Biodefense and Emerging Infectious Diseases, University of Texas Medical Branch, Galveston, Texas, USA; The University of Tennessee Knoxville, Knoxville, Tennessee, USA

**Keywords:** bacteriophage, therapeutic, *Yersinia pestis*, Tier-1 select agent, pneumonic plague, rat model, aerosol challenge, biodefense

## Abstract

*Yersinia pestis,* one of the deadliest bacterial pathogens ever known, is responsible for three plague pandemics and several epidemics, with over 200 million deaths during recorded history. Due to high genomic plasticity, *Y. pestis* is amenable to genetic mutations as well as genetic engineering that can lead to the emergence or intentional development of pan-drug-resistant strains. Indeed, antibiotic-resistant strains (e.g., strains carrying multidrug-resistant or MDR plasmids) have been isolated in various countries and endemic areas. Thus, there is an urgent need to develop novel, safe, and effective treatment approaches for managing *Y. pestis* infections. This includes infections by antigenically distinct strains for which vaccines (none FDA approved yet) may not be effective and those that cannot be managed by currently available antibiotics. Lytic bacteriophages provide one such alternative approach. In this study, we examined post-exposure efficacy of a bacteriophage cocktail, YPP-401, to combat pneumonic plague caused by *Y. pestis* CO92. YPP-401 is a four-phage preparation effective against a panel of at least 68 genetically diverse *Y. pestis* strains. Using a pneumonic plague aerosol challenge model in gender-balanced Brown Norway rats, YPP-401 demonstrated ~88% protection when delivered 18 h post-exposure for each of two administration routes (i.e., intraperitoneal and intranasal) in a dose-dependent manner. Our studies provide proof-of-concept that YPP-401 could be an innovative, safe, and effective approach for managing *Y. pestis* infections, including those caused by naturally occurring or intentionally developed multidrug-resistant strains.

**IMPORTANCE:**

Currently, there are no FDA-approved plague vaccines. Since antibiotic-resistant strains of *Y. pestis* have emerged or are being intentionally developed to be used as a biothreat agent, new treatment modalities are direly needed. Phage therapy provides a viable option against potentially antibiotic-resistant strains. Additionally, phages are nontoxic and have been approved by the FDA for use in the food industry. Our study provides the first evidence of the protective effect of a cocktail of four phages against pneumonic plague, the most severe form of disease. When treatment was initiated 18 h post infection by either the intranasal or intraperitoneal route in Brown Norway rats, up to 87.5% protection was observed. The phage cocktail had a minimal impact on a representative human microbiome panel, unlike antibiotics. This study provides strong proof-of-concept data for the further development of phage-based therapy to treat plague.

## INTRODUCTION

*Yersinia pestis* is a gram-negative, non-motile, coccobacillus bacterium, and human infections can lead to three deadly forms of plague, which is one of the oldest diseases responsible for the death of over 200 million people in recorded history (1, 2). Likely because of the deadly combination of high fatality and contagiousness associated with pneumonic plague, *Y. pestis* was the first bacterium used as a biological warfare agent when the Tatars catapulted plague-infected corpses into the sieged Black Sea port of Kaffa (currently Feodossia, Ukraine) in 1346 (3, 4). During World War II, the Japanese released *Y. pestis*-infected fleas to infect humans in several Chinese cities, thus causing small epidemics of plague (5). Consequently, *Y. pestis* was the key bacterium for many bioweapon development programs in the United States, the former Soviet Union (FSU) (6), and other countries. Accordingly, the U.S. Centers for Disease Control and Prevention (CDC) and National Institute of Allergy and Infectious Diseases (NIAID) classified a handful of bacteria (including *Y. pestis*) and viruses as category “A” select agents (7–9). *Y. pestis* now belongs to a short list of Tier-1 select agents because of its very high propensity to impact public health with mass casualties as a result of person-to-person transmission during pneumonic plague. While the incidence of plague has declined during the last several decades, cases of *Y. pestis* infections are regularly registered globally each year (10, 11). In the United States, in 2015, 15 human cases of plague were reported, resulting in four deaths; in 2017, the island of Madagascar experienced a large outbreak, where approximately 2,350 cases of plague (~70% in pneumonic form) occurred, including 202 deaths (2).

Current prevention and treatment modalities for *Y. pestis* infections are suboptimal at best. The current plague vaccines in preclinical development or the earlier versions of vaccines, with none approved yet by the U.S. Food and Drug Administration (FDA), have variable efficacy, limited antigenic complexity, require regular boosting, and/or have severe side effects (2, 12, 13). A limited number of clinical trials have been performed based on subunit vaccines containing only the capsular antigen F1 and the low-calcium response V antigen (LcrV), with correlates of protection still undefined (14, 15). Because of the absence of a safe and effective *Y. pestis* vaccine, antibiotics remain the last bastion of defense against a possible *Y. pestis* attack or during an epidemic. However, because of high genomic plasticity (16), *Y. pestis* is amenable to genetic engineering that can lead to the emergence of pan-drug-resistant strains, which would be very difficult, if not impossible, to manage using currently available antibiotics. One example of such engineering is the highly virulent, antibiotic-resistant F1^−^ strain(s) developed at the Institute of Applied Microbiology (located in Obolensk, Russia) (17). Other antibiotic-resistant mutants may emerge or be similarly engineered by other countries and/or terrorist organizations, which could be intentionally or unintentionally released. The consequences associated with the dissemination of *Y. pestis* strains that are untreatable with antibiotics could be catastrophic. Thus, there is a critical need to develop novel, safe, and effective treatment approaches for managing *Y. pestis*-associated diseases, including those caused by antigenically distinct strains (against which vaccines may not be optimally effective) and by those that cannot be killed by available antibiotics. Lytic bacteriophages can provide one such alternative approach.

Bacteriophages (or phages) are viruses that target bacterial hosts. They were identified during the early part of the 20th century by Felix d’Herelle (18, 19) and were first used successfully to treat bubonic plague in 1925 (20). Phages are the oldest (~3 billion years old) and most ubiquitous organisms (~10^30^–10^31^ phage particles) on Earth. Lytic phages are very effective at killing their targeted host bacteria and, in contrast to antibiotics, they are very specific (i.e., phages will lyse related strains or a subgroup of strains, but will not lyse strains of other, unrelated bacteria). During the early 20th century, their remarkable antibacterial activity prompted the use of phages for treating many bacterial diseases, but for various reasons [reviewed in reference (21)], their use gradually declined in the West after antibiotics became widely available. However, the emergence of multi-antibiotic-resistant pathogens has rekindled interest in the possible therapeutic applications of bacteriophages. In this context, the mechanisms by which antibiotics and lytic phages kill bacteria (and the mechanisms of bacterial resistance to antibiotics vs phages) are fundamentally different (22). As a result, lytic phages can kill bacteria that cannot be eliminated by currently available antibiotics. Furthermore, the development of an antibiotic resistance does not impact the lytic potency of phages and vice versa. Thus, lytic phages may provide a critical complementing modality to antibiotics, for preventing and/or treating diseases caused by multi-antibiotic-resistant bacteria.

Several previous studies have shown *in vitro* phage efficacy against *Y. pestis* (23–25). Only a few studies have demonstrated *in vivo* efficacy (26, 27). In one study, phage treatment was shown to delay the progression of pneumonic plague; however, all mice still succumbed unless antibiotic therapy was co-administered (27). Here, we assessed a four-phage preparation, YPP-401, to target *Y. pestis in vitro* as well as *in vivo* utilizing our previously well-characterized Brown Norway rat model of pneumonic plague infection that demonstrates 100% mortality (28). These proof-of-concept studies demonstrate the high specificity of YPP-401 for *Yersinia* spp. and its utility as a post-exposure therapeutic.

## RESULTS

### The bacteriophage cocktail YPP-401 and its component monophages demonstrate host-specific lytic activity *in vitro*

A proprietary bacteriophage cocktail formulation, YPP-401, containing four monophages (i.e., YPIX-2, -4, -5, and -6) was developed and characterized using a combination of previously identified phages specific for *Y. pestis* (24, 29). The YPP-401 component monophages previously were also shown to be effective against all tested high containment/select agent *Y. pestis* strains (24, 29).

Here, we assessed the ability of the YPP-401 formulation to target BSL-2 *Yersinia* spp. (Table 1) and non-*Yersinia* strains (Table S1; Fig. S1) *in vitro* using a combination of liquid and/or spot test (30) assays at various phage concentrations and bacterial growth conditions. Additionally, the virulent wild-type *Y. pestis* CO92 strain (i.e., the challenge strain) was susceptible to the YPP-401 cocktail along with all tested *Y. pestis* and *Y. pseudotuberculosis* strains (Table 1). Some *Y. pestis* phages have been reported to have activity in laboratory medium but not blood (23); therefore, we also performed the plate-based assays for *Y. pestis* CO92 susceptibility on blood agar plates and obtained similar results (data not shown). Only one *Y*. *aldovae* strain was susceptible to the YPP-401 formulation, while strains representing four other *Yersinia* spp. (i.e., *Y. enterocolitica*, *Y. frederiksenii*, *Y. kristensenii*, and *Y. mollaretii*) were not susceptible (Table 1). Additionally, to demonstrate the specificity of YPP-401 for *Yersinia* spp., susceptibility to the YPP-401 cocktail was evaluated against a representative panel of bacteria from the human microbiome (Table S1; Fig. S1). All of the non-*Yersinia* spp. tested were not susceptible to YPP-401 with the exception of several *Escherichia coli* strains. As expected, YPP-401 was highly specific for *Yersinia* spp. compared to antibiotic treatments, including ciprofloxacin, a first-line antibiotic approved for the treatment of plague that broadly inhibited the growth of various bacteria (Fig. S1).

**TABLE 1:** *In vitro* lytic activity of YPP-401 against *Yersinia* spp.^*a*^

Genus	Species	Strain	Susceptible^*b*^
*Yersinia*	*aldovae*	ATCC 35236	+
*Yersinia*	*aldovae*	CNY 7112	−
*Yersinia*	*enterocolitica*	30.42.67	−
*Yersinia*	*enterocolitica*	8081	−
*Yersinia*	*frederiksenii*	CNY 867	−
*Yersinia*	*frederiksenii*	WA 935	−
*Yersinia*	*kristensenii*	ATCC 33638	−
*Yersinia*	*kristensenii*	WE 180/98	−
*Yersinia*	*mollaretii*	WE 149/92	−
*Yersinia*	*mollaretii*	WS 43/94	−
*Yersinia*	*pestis*	A12 D6	+
*Yersinia*	*pestis*	A1122	+
***Yersinia***	***pestis***	**CO92**	**+**
*Yersinia*	*pestis*	Harbin 35	+
*Yersinia*	*pestis*	K25 D80	+
*Yersinia*	*pestis*	KIM D2	+
*Yersinia*	*pestis*	KIM D19	+
*Yersinia*	*pestis*	KIM D23	+
*Yersinia*	*pestis*	KIM D27	+
*Yersinia*	*pestis*	Kimberly D13	+
*Yersinia*	*pestis*	KUMA D8	+
*Yersinia*	*pestis*	TS D4	+
*Yersinia*	*pestis*	Yokohama D11	+
*Yersinia*	*pseudotuberculosis*	ATCC 23207	+
*Yersinia*	*pseudotuberculosis*	ATCC 29833	+
*Yersinia*	*pseudotuberculosis*	CDC 542–84	+
*Yersinia*	*pseudotuberculosis*	CDC 801–84	+

^*a*^ BOLD, virulent, wild-type *Y. pestis* strain used in challenge studies.

^*b*^ Susceptibility tested across various phage concentrations in liquid and/or solid spot test assays; ‘+’ denotes the indicated strain was susceptible to YPP-401, while ‘-’ denotes the strain was not susceptible.

### YPP-401 provides protective efficacy *in vivo* when treatment is initiated at 18 h post-exposure

**Fig 1:**
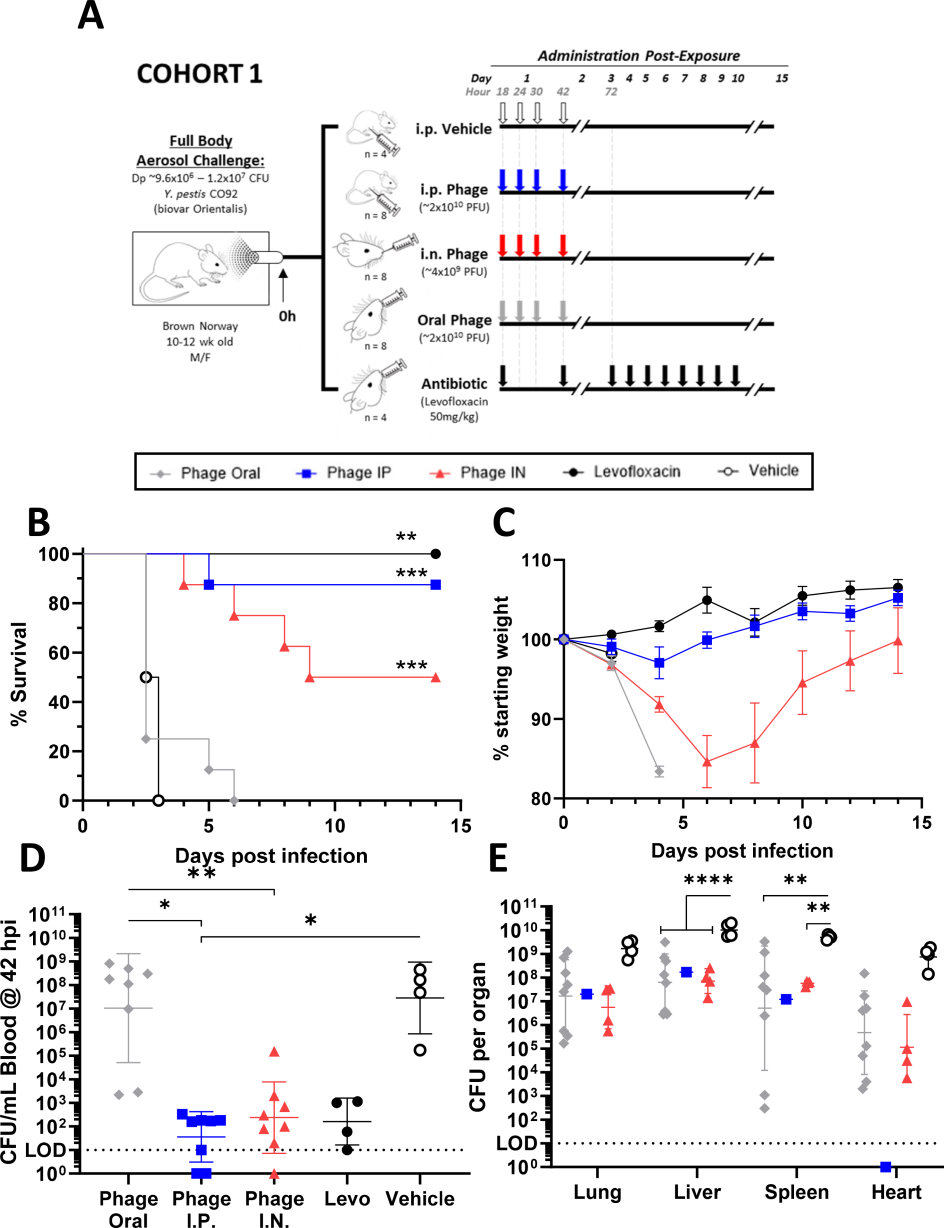
YPP-401 provides post-exposure efficacy against *Y. pestis* in rats when delivered intraperitoneally or intranasally at 18 hpi. *COHORT 1*: (**A**) Schematic of study design and dosing regimens. Shown are (**B**) survival, (**C**) body weight, and (**D**) *Y. pestis* burden in the blood at 42 hpi (taken immediately prior to 42 h treatment timepoint). Other measures were (**E**) terminal organ bacterial burden (i.e., lung, liver, heart, spleen) and clinical score (i.e., appearance, activity, respirations, facial expression; Table S2). Brown Norway rats (M/F; 10–12 wk) received a full body aerosol challenge (0 h) of WT virulent *Y. pestis* strain CO92 at Dp ~ 9.56 × 10^6^ to 1.24 × 10^7^ CFU. At 18 hpi, cohort 1 received either 2 days (total four doses, BID, ~q6–12 h) of i.p. vehicle (white/open circles; PBS; *n* = 4), i.p. YPP-401 (blue squares; ~2 × 10^10^ PFUs; *n* = 8), i.n. YPP-401 (red triangles; ~4 × 10^9^ PFUs; *n* = 8), oral YPP-401 (gray diamonds; ~2 × 10^10^ PFUs; *n* = 8), or 10 oral QD doses of levofloxacin (levo) (black circles; 50 mg/kg; *n* = 4). Body weight graph (**B**) shows the arithmetic mean with the standard error of the mean (SEM) depicted as error bars. Bacteremia (**D**) and organ burden (**E**) show the geometric mean with geometric standard deviation (GSD) shown as error bars. For statistical comparisons, Kaplan-Meier analysis with Logrank (Mantel-Cox) test was used for survival (**B**), one-way analysis of variance (ANOVA) with Kruskal-Wallis test and Dunn’s multiple comparison test were used for (**D**) while two-way ANOVA with Tukey’s post-hoc test was used for (**E**). Limit of detection (LOD) is depicted by the dotted horizontal line. Studies were performed at UTMB under ABSL-3. The asterisk indicates a significant difference compared to vehicle control or between the two groups indicated by a horizontal line. *P < 0.05; **P < 0.01; ***P < 0.001; ****P < 0.0001.

Previous studies conducted by our laboratory using the aerosol challenge model of pneumonic plague in Brown Norway rats implanted with telemetry showed that the febrile response (≥39°C temperature for at least 1 h of duration) usually started after 36 hpi (unpublished data). Here, animals in cohort 1 were subjected to a full-body aerosol challenge [*Y. pestis* CO92 with a presented dose (Dp) of 9.56 × 10^6^ to 1.24 × 10^7^ CFU] followed by a YPP-401 treatment regimen starting at 18 hpi as depicted in Fig. 1A. YPP-401 treatment was administered by a variety of routes including intraperitoneal (i.p.), intranasal (i.n.), oral (p.o.), and intramuscular (i.m.). At 18 hpi, cohort 1 animals were administered four total doses of YPP-401 over 24–42 h approximately every 6–12 h (i.e., 18, 24, 30, and 42 hpi) by one of three separate routes: oral (~2 × 10^10^ plaque-forming units [PFU]/dose), i.n. (~4 × 10^9^ PFU/dose), or i.p. (~2 × 10^10^ PFU/dose) (Fig. 1A). Control treatment groups included vehicle alone [i.e., phosphate-buffered saline (PBS)] delivered i.p. four times at the same intervals as YPP-401, and levofloxacin (50 mg/kg), which was delivered orally, daily for 10 days. Survival data for cohort 1 is shown in Fig. 1B. Rats treated orally with YPP-401 succumbed to infection within 2–6 days with no significant difference in survival compared to vehicle controls although two animals demonstrated a delayed time to death compared to controls. Intraperitoneal treatment with YPP-401 resulted in 87.5% survival (*P* < 0.001 compared to vehicle) with one rat succumbing to the disease at day 5 post-infection (dpi). Intranasal treatment with YPP-401 resulted in 50% survival (*P* < 0.001 compared to vehicle), with rats succumbing to infection on 4–9 dpi with two males and two females each succumbing in this treatment group indicating a lack of sex-based differences in therapeutic effectiveness. Due to administration volume limitations for i.n. delivery, animals received 1/5th of the YPP-401 dose than that delivered by the i.p. route. All vehicle treated rats succumbed to infection by 3 dpi. Rats treated with levofloxacin, the positive antibiotic control, had 100% survival (*P* < 0.05 compared to vehicle) at 14 dpi after 10 daily doses of the antibiotic.

### Clinical scores and bacterial burden in blood and organs correlate with survival in YPP-401 treated animals

Clinical signs and symptoms were assessed and included weight loss (Fig. 1C) and clinical scores (Table S2) that accounted for independent variables for appearance, activity level, respiration, and facial expression. In general, across all groups, clinical scores correlated with time to death (Table S2). Additionally, in rats receiving i.n. YPP-401, 7 out of 8 rats lost 15%–20% bodyweight with peak weight loss occurring by 6–8 dpi. Surviving animals began recovering weight by 6–10 dpi (Fig. 1C). In contrast, a significant weight loss in the i.p. YPP-401 treated rats was only observed in the single animal that succumbed to the disease at 5 dpi with a body weight loss of 15.3% at the last measured timepoint prior to succumbing to disease (Fig. 1C). These results are consistent with our previous studies (unpublished data) where we typically did not observe significant weight loss until 4 dpi and, thus, explains the minimally observed weight loss in the vehicle- and YPP-401 orally-treated animals that succumbed to disease within 4 dpi (Fig. 1B).

To determine the effect of YPP-401 treatment on bacteremia, blood was collected at 42 hpi (i.e., immediately before the last phage dose) and titrated on SBA plates (Fig. 1D; Table S2). Groups with low survival rates (i.e., vehicle and oral YPP-401) had high levels of *Y. pestis* in the blood with an average of 10^8^ CFU/mL (Fig. 1D). Groups treated with three doses of YPP-401 by i.p. or i.n. had significantly lower levels of *Y. pestis* in the blood than animals treated orally (Fig. 1D). Additionally, bacteremia was significantly lower in i.p. YPP-401 treated animals than vehicle treated rats. Intraperitoneal YPP-401 treated rats had similar levels of *Y. pestis* bacteremia as the levofloxacin treated controls (Fig. 1D). Two animals treated orally with YPP-401 had significantly lower levels of *Y. pestis* in the blood and correlated with delayed time to death (Table S2). In the one i.p. YPP-401 treated animal that succumbed to disease, only 180 CFU/mL of blood were detectable and there was a delayed time to death (Table S2).

Total organ bacterial burden also was evaluated in post-treatment animals that succumbed to disease. Bacterial loads in the terminal lungs, liver, spleen, and heart were quantitated from tissue homogenates on SBA plates (Fig. 1E; Table S2). As expected, the vehicle treated rats had the highest bacterial load in all four organs analyzed. Rats treated with YPP-401 that succumbed, regardless of the administration route, had lower levels of *Y. pestis* in the lungs, liver, and spleen compared to the vehicle controls and generally had similar levels among all YPP-401 treated groups (Fig. 1E). The organ burden in oral, i.p., or i.n. YPP-401 treated animals was statistically significant for the liver compared to vehicle controls, while in the spleen, only oral and i.n. YPP-401 treated animals were statistically significant. The oral and i.n. YPP-401 treated groups had similar levels of *Y. pestis* in the heart. In general, compared to animals that succumbed to disease, YPP-401 treated rats had statistically lower levels of *Y. pestis* in blood and organs compared to the vehicle control (Fig. 1D and E, respectively; Table S2).

### YPP-401 provides dose-dependent protective efficacy and reduced bacterial loads *in vivo* when delivered intranasally beginning at 18 h post-exposure

To determine whether increasing the i.n. YPP-401 dose could improve post-exposure protection when delivered starting at 18 hpi, cohort 2 animals were challenged with *Y. pestis* CO92 by the aerosol route as described above for cohort 1 (Fig. 1A). Here, rats treated with four total doses of YPP-401 (between 18 and 42 hpi) were given approximately 5× the dose (i.e., ~2 × 10^10^ PFU/dose) delivered in cohort 1 (i.e., ~4 × 10^9^ PFU/dose). As in cohort 1, rats were randomly distributed into four aerosol challenge runs where the presented dose between runs ranged from 9.79 × 10^6^ to 1.06 × 10^7^ CFUs.

**Fig 2:**
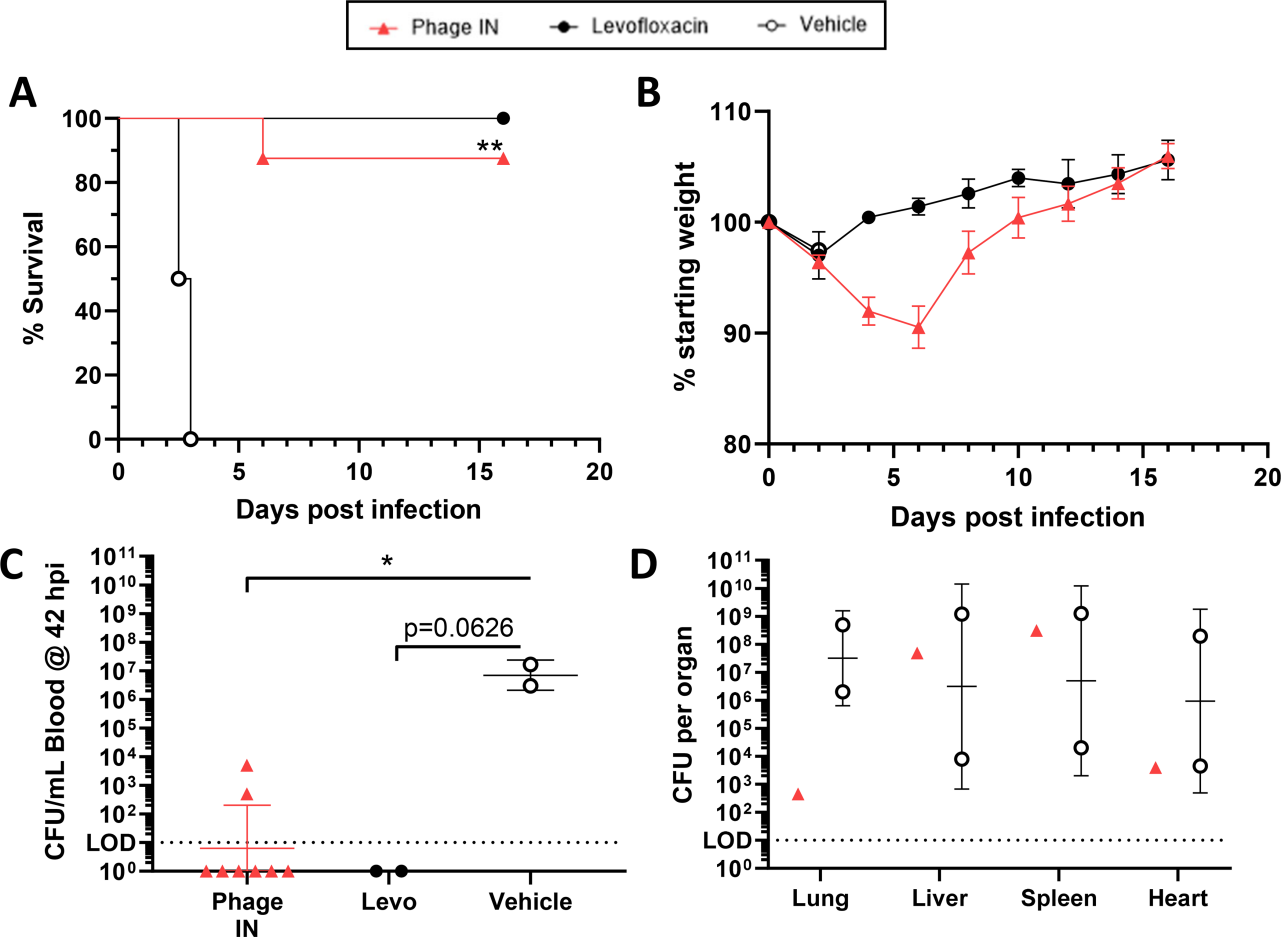
YPP-401 provides dose-dependent post-exposure efficacy against *Y. pestis* in rats when delivered intranasally at 18 hpi. *COHORT 2*: Animals were challenged as described in Fig. 1A. Similar dosing regimens starting at 18 hpi were used with the following changes: cohort 2 received either 2 days (total four doses, BID, ~q6–12 h) of i.p. vehicle (white/open circles; PBS; *n* = 2), i.n. YPP-401 (red triangles; ~2 × 10^10^ PFUs; *n* = 8), or 10 oral QD doses of levo (black circles; 50 mg/kg; *n* = 2). Shown are (**A**) survival, (**B**) body weights, and (**C**) *Y. pestis* burden in the blood at 42 hpi (taken immediately prior to 42 h treatment timepoint). Other measures were (**D**) terminal organ bacterial burden (i.e., lung, liver, heart, spleen) and clinical score (i.e., appearance, activity, respirations, facial expression; Table S3). Body weight graph (**B**) shows the arithmetic mean with the SEM depicted as error bars. Bacteremia (**C**) and organ burden (**D**) show the geometric mean with GSD shown as error bars. For statistical comparisons, Kaplan-Meier analysis with Logrank (Mantel-Cox) test was used for survival (**A**), one-way ANOVA with Kruskal-Wallis test and Dunn’s multiple comparison test were used for (**C**) while two-way ANOVA with Tukey’s post-hoc test was used for (**D**). LOD is depicted by the dotted horizontal line. Studies were performed at UTMB under ABSL-3. The asterisk indicates a significant difference compared to vehicle control or between the two groups indicated by a horizontal line. *P < 0.05; **P < 0.01.

Figure 2A shows that increasing the YPP-401 i.n. dose increased survival from 50% in cohort 1 (Fig. 1B) to 87.5% (Fig. 2A). Control groups behaved as expected with all vehicle treated animals succumbing by 3 dpi and all levofloxacin treated rats, which received 10 daily doses, surviving through 14 dpi (Fig. 2A). These results, interestingly, demonstrate that i.n. YPP-401 treatment can be as effective as i.p. YPP-401 treatment when administered at the same dose. Cohort 2 rats treated with YPP-401 i.n. lost no more than ~10% body weight (Fig. 2B) compared to cohort 1 YPP-401 i.n. treated animals (Fig. 1C), with peak weight loss observed at 6 dpi, and all surviving rats gaining weight by day 8 (Fig. 2B). Levofloxacin treated rats lost minimal weight with one rat losing 5% body weight by 2 dpi, which recovered to pre-infection levels by 4 dpi (Fig. 2B).

As performed for cohort 1 above, bacteremia (Fig. 2C; Table S3) and organ burden (Fig. 2D; Table S3) were assessed at either 42 hpi (prior to treatment) or terminally, respectively. Seventy-five percent (6/8) of YPP-401 i.n. treated rats had no detectable *Y. pestis* in the blood (Fig. 2C; Table S3). The two animals with detectable bacteria in the blood had significantly reduced levels (i.e., <10^4^ CFU/mL) compared to the vehicle controls (i.e., >10^6^ CFU/mL; Fig. 2C) and were similar to levels in cohort 1 animals treated with levofloxacin or YPP-401 delivered by i.n. or i.p. routes (Fig. 1D). The cohort 2 levofloxacin treated rats (*n* = 2) had no detectable *Y. pestis* in the blood. The total organ burden in cohort 2 rats that succumbed was evaluated; however, due to the low number of rats that succumbed in the treatment group, statistical methods could not be applied. *Y. pestis* found in the lungs of the only terminal YPP-401 i.n. treated rat was 4–6 logs lower than vehicle treated rats (Fig. 2D), while no statistically significant conclusion could be reached for bacterial burden in the liver, spleen, and heart compared to the vehicle controls due to the low number of animals (*n* = 2) and high variability in the vehicle control group (Fig. 2D).

### Highly protective YPP-401 dosing regimens initiated at 18 h post-exposure are not sufficient to protect against *Y. pestis* challenge at a more delayed time post-exposure when clinical signs and symptoms are apparent

**Fig 3:**
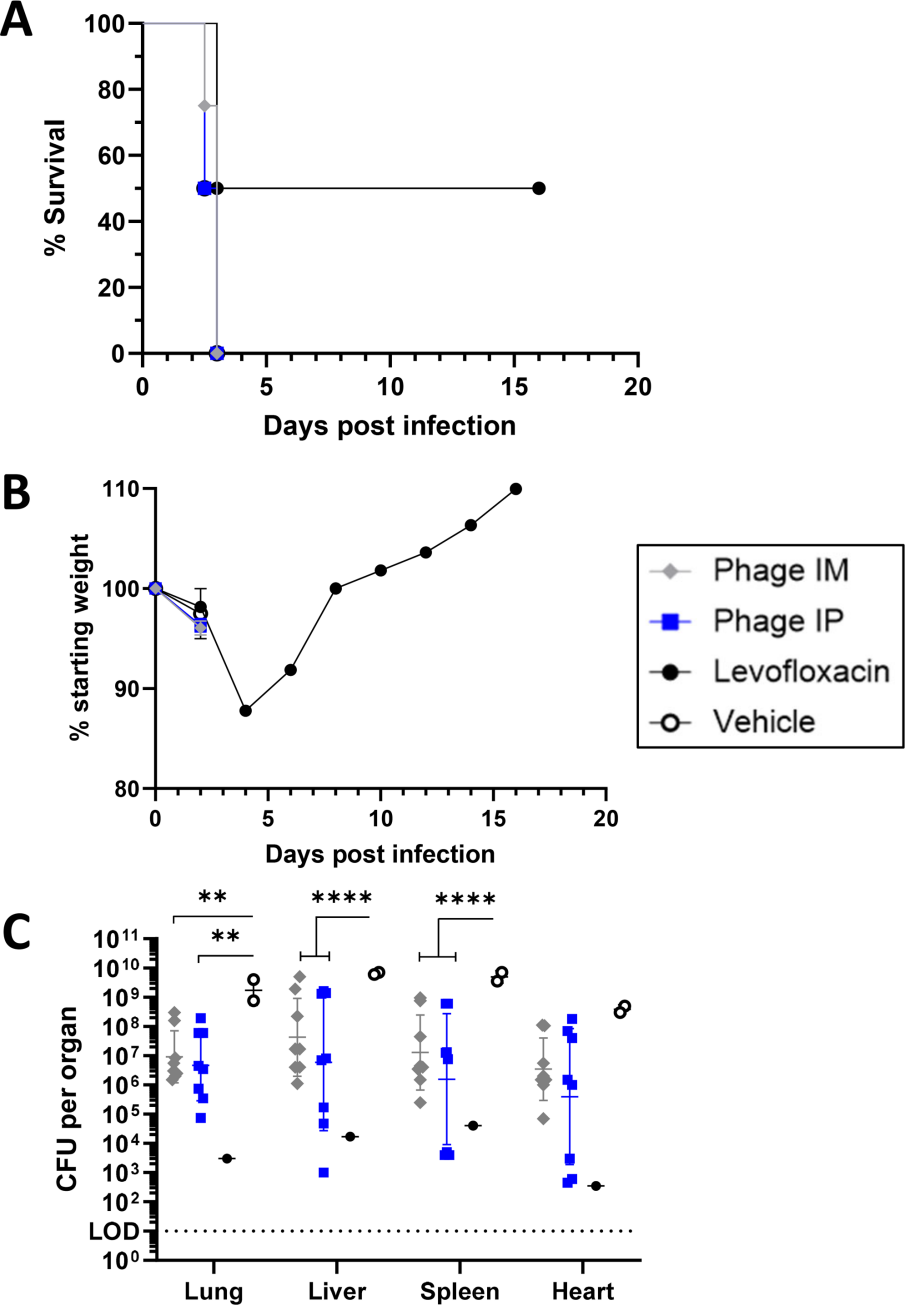
YPP-401 dosing regimens protective at 18 hpi are insufficient when initiated at 42 hpi. *COHORT 3*: Animals were challenged as described in Fig. 1A. Similar dosing regimens were started at 42 hpi with the following changes: cohort 3 received either one or two doses (BID, ~q6–12 h) of i.p. vehicle (white/open circles; PBS; *n* = 2), i.m. YPP-401 (gray circles; ~5 × 10^10^ PFUs; *n* = 8), i.p. YPP-401 (blue squares; ~5 × 10^10^ PFUs; *n* = 8), or 1 or 10 QD doses of levo (black circles; 50 mg/kg; *n* = 2). Shown are (**A**) survival, (**B**) body weights, and (**C**) *Y. pestis* burden in terminal organs (i.e., lung, liver, heart, spleen) and clinical score (i.e., appearance, activity, respirations, facial expression; Table S4). Body weight graph (**B**) shows the arithmetic mean with the SEM depicted as error bars. Organ burden (**C**) shows the geometric mean with GSD shown as error bars. For statistical comparisons, Kaplan-Meier analysis with Logrank (Mantel-Cox) test was used for survival (**A**), two-way ANOVA with Tukey’s post-hoc test was used for (**D**). LOD is depicted by the dotted horizontal line. Studies were performed at UTMB under ABSL-3. The asterisk indicates a significant difference between the two groups indicated by a horizontal line. *P < 0.05; **P < 0.01; ***P < 0.001; ****P < 0.0001.

To determine whether the YPP-401 i.p. protective post-exposure dosing regimen used in cohort 1 (Fig. 1A) is sufficient to protect animals at a more delayed time post-exposure, a third cohort (cohort 3) was treated starting at 42 hpi after *Y. pestis* CO92 aerosol challenge. It should be noted, as has been shown previously, that at 42 hpi, clinical signs and symptoms of the disease are typically apparent and even frontline antibiotic therapy initiated at this timepoint has much reduced efficacy (31, 32). As shown in Fig. 3A, rats treated with YPP-401 i.p. starting at 42 hpi, planned to be administered 6–12 h apart, were only able to receive one or two doses (Table S4) of YPP-401 before succumbing to disease, and, thus, were ineffective at providing protection under these conditions. Moreover, even the levofloxacin control treatment, which reliably rescues 100% of treated rats when administered at 18 hpi, was only effective in 50% of the animals (*n* = 2) with one rat succumbing to the infection having received 1 out of the 10 intended daily doses (Fig. 3A; Table S4). Under emergency or mass treatment settings, i.p. administration is not practical; therefore, we also assessed whether YPP-401 delivered intramuscularly (i.m.) could provide post-exposure protection under these delayed and stringent conditions where treatment was initiated at 42 hpi. As seen with YPP-401 delivered i.p., i.m. administration did not improve protection under this dosing regimen (Fig. 3A; Table S4).

Prior to treatment at 42 hpi, blood was collected to evaluate levels of bacteremia. All rats had high levels (up to 10^7^ CFU/mL) of *Y. pestis* in the blood (Table S4). Rats that succumbed were necropsied to assess the levels of *Y. pestis* present in the lungs, liver, spleen, and heart. While vehicle treated rats had uniformly high levels of *Y. pestis* in these organs at levels > 10^8^ CFU/organ, the number of *Y. pestis* found in YPP-401 or levofloxacin treated rats was more varied (Fig. 3C; Table S4) likely due to killing by either phage or antibiotics, respectively. The single levofloxacin treated rat that survived showed enhanced morbidity over that seen in cohorts 1 and 2 with >12.5% peak body weight loss by 4 dpi with recovery occurring by 6 dpi (Fig. 3B). Table S4 shows the values for individual rats that succumbed to infection (e.g., the number of treatment doses received, bacteremia, organ burden, clinical score before succumbing to disease, and time to death).

## DISCUSSION

### *Yersinia pestis*, a potential biotreat

The diseases *Yersinia pestis* cause have a high fatality rate; of approximately 30% for bubonic plague, while septicemic and pneumonic plague, if not treated within 24 h of symptom onset, have mortality rates approaching 100% (33). The bacteria can spread rapidly person to person via the aerosol route, which makes containment difficult. A 2017 outbreak of plague in Madagascar was notable because, while historically most of the cases during an outbreak are bubonic, during the Madagascar epidemic over 70% of cases were pneumonic (34). The consequences of similar outbreaks will be even more destructive if it is caused by an MDR strain (e.g., naturally mutated or genetically engineered to be resistant to all currently available antibiotics by a terrorist organization or rogue nation). Thus, we may lack effective tools for preventing and/or treating the diseases the organism causes. In this context, although MDR has been relatively rare in *Y. pestis*, isolates of *Y. pestis* showing tetracycline resistance and reduced susceptibility to streptomycin have been described in some African countries and China. Likewise, MDR isolates have been reported from Madagascar, and a strain resistant to most of the antibiotics commonly used in prophylaxis and therapy of plague was isolated in Mongolia (35–38). Moreover, several countries, including the FSU, used to have bioweapons programs designed to engineer *Y. pestis* clones that would be more difficult to treat with antibiotics and could also be better aerosolized for even larger and more rapid spread (6).

### Phage therapy, a promising approach to mitigate bacterial infections

There are multiple favorable qualities in support of therapeutic phages. Phages, in contrast to antibiotics and other antimicrobial agents, are extremely host specific (i.e., they attack specific bacterial strains or subgroups of strains) and, thus, have minimal potential for the disruption of normal or commensal flora. Lytic phages are bactericidal, as opposed to bacteriostatic, thereby possibly providing faster and unreversible killing of the pathogen. The mechanism of action for phages is independent from traditional antimicrobial mechanisms and thereby can be used against MDR strains (39, 40). Finally, as compared to many vaccines in development, there is a long history of lack of toxicity with phages, and they have even been used in severely immunodeficient humans (41–43).

### YPP-401 is a potent phage-based product that targets *Y. pestis*

We have identified a candidate *Y. pestis* phage cocktail preparation, YPP-401. Using PhageSelector, we identified the optimal combination of *Y. pestis* phages to be included in our candidate YPP-401 preparation. This potent phage cocktail contains four lytic bacteriophages with excellent lytic potency against all tested *Y. pestis* strains [Table 1 and (24, 29)], as determined using the classical Spot Test method (44). This collection includes geographically (including the USA, Iran, Georgia, Azerbaijan, Armenia, Africa, and Japan) and temporally (over >30 years) distinct strains collected from various sources (e.g., humans, rodents, fleas, soil).

The potential impact of YPP-401 phage therapy on the microbiome was assessed (Fig. S1) and was compared to various antibiotics, which have a broad spectrum of antimicrobial activity. Phages are considered narrow spectrum antimicrobial agents, where specificity is often limited to the genus and/or species level. This study confirmed the specificity of YPP-401 for *Yersinia* spp. YPP-401 is able to lyse a wide variety of *Y. pestis* strains as well as some non-*pestis Yersinia* spp. including *Y. pseudotuberculosis* and *Y. aldovae* (Table 1). Cross reactivity of phages between *Y. pestis* and *Y. pseudotuberculosis* has previously been reported in the literature (25). Given that *Y. pestis* is believed to have evolved from *Y. pseudotuberculosis* as few as 6,000 years ago, this result is not that surprising. Similarly, *Y. aldovae* was previously classified as *Y. enterocolitica* X2 group but was classified as a distinct species in 1984. Very little has been published about *Y. aldovae,* but they are generally isolated from water and are considered non-pathogenic. Several strains of *E. coli* were also noted to be susceptible to YPP-401 (Table S1; Fig. S1). Although not closely related, *Escherichia* and *Yersinia* are both genera within the *Enterobacterales* order. The ability of *Y. pestis* phages to lyse *E. coli* (25) as well as similarity to known *E. coli* phages (45) has been previously reported. Previous studies have characterized receptors used by various *Yersinia* phages to infect the bacterial cell. The most commonly used are various regions of LPS and outer membrane proteins, such as Ail (attachment invasion locus), OmpA (outer membrane protein A), and OmpF (46–50). Mutations in LPS regions, in addition to Ail, have been identified as potential targets for phage resistance (51).

The LPS between *Y. pestis* and *E. coli* is significantly different as *Y. pestis* has rough LPS, which lacks the O antigen. In addition, a change from tetra-acylated lipid A at 37°C to hexa-acylated lipid A at 21–27°C is the characteristic trait for all *yersiniae*. The core section of LPS is more conserved between *Y. pestis* and *E. coli*. Sequence alignment of LPS core biosynthesis gene WaaA between *E. coli* K12 and *Y. pestis* CO92 exhibits 79.8% identity between them. The homology of the phage receptors, Ail, OmpA, and OmpF, between *Y. pestis* and *E. coli* is ~24%–34%, 77%, and 58%, respectively. Therefore, it is possible that bacteriophages use similar receptors for the entry of both *Y. pestis* and *E. coli.*

For example, *Yersinia* φA1122 is a member of the T7 phage family which is well studied in *E. coli*. It has been shown that φA1122 binds to the core section of LPS similar to other T7 family phages, including those that infect other *Yersinia* and *E. coli* which all use LPS for entry (48). Furthermore, the phage tail structures of φA1122 and *E. coli* phage T3 (also a T7 family member) are very similar, 98.9% amino acid identity with six different amino acids between the two phage tails (52). As expected, YPP-401 had no activity against gram-positive members of the representative microbiota (Table S1) in contrast with the antibiotics tested (Fig. S1). This suggests that YPP-401 phage therapy is unlikely to cause significant changes in the host microbiome during treatment.

### YPP-401 provides strong post-exposure protection in a rat challenge model

While several published studies have shown phages can target *Y. pestis in vitro*, only a few studies have examined *in vivo* efficacy of *Y. pestis* phages (23, 24, 27). To this end, we examined whether YPP-401 could provide post-exposure protection at “early” (i.e., 18 hpi; Fig. 1 and 2) or “late” (i.e., 42 hpi; Fig. 3) times post-lethal challenge with *Y. pestis* CO92 using different dosing regimens, varying route (i.e., i.p., i.n., p.o., i.m.), and/or YPP-401 dose (~4 × 10^9^–5 × 10^10^ PFU/dose) in a previously characterized, 100% lethal rat aerosol challenge model (28, 32, 53–55).

In cohort 1 (Fig. 1A), rats received a “low” dose of YPP-401 (~2 × 10^10^ PFU/dose by i.p. or p.o. or ~4 × 10^9^ PFU/dose by i.n.—dose varied due to maximum amount of volume delivered per route) starting at 18 h post-challenge. YPP-401 was administered about every (q) 6–12 h for a total of 4 doses until 42 hpi, and the time at which animals typically become symptomatic. Control groups included vehicle alone or levofloxacin (levo; 50 mg/kg), a first line antibiotic approved for the treatment of pneumonic plague, administered p.o. daily (i.e., QD) for 10 days and previously known to fully protect in this model. Rats receiving low dose YPP-401 by i.p. had an 88% survival rate, while those treated by i.n. had a 50% rate of survival (Fig. 1B). Despite a slight delay in mortality, those receiving YPP-401 p.o. succumbed. Rats receiving YPP-401 i.p. had similar levels of *Y. pestis* in their blood as the levofloxacin controls at 42 hpi (Fig. 1D). Bioavailability of phage by the oral route is likely poor. In an earlier study (56), it was reported that while orally administered phages could be detected in the blood and liver, there was a 4-log fewer number of phages compared to the numbers administered (56). Bioavailability has also been compared between i.p. and i.n. phage therapy. While i.p. administration of phages led to their higher numbers in the spleens of treated animals, i.n. therapy resulted in more phages in the lungs by 3–4 logs (27). It should be noted that during organ collection, hearts were not perfused, so the bacterial load quantified in the heart might reflect bacteremia as well as colonization of the heart itself. Furthermore, the heart is not commonly examined in plague studies. In contrast, bioburden in the lung, liver, and spleen has been extensively reported in the literature for plague studies.

To determine whether i.n. YPP-401 post-exposure protection could be improved by increasing the YPP-401 dose, rats in cohort 2 received an intermediate dose (~2 × 10^10^ PFU/dose, ~5× the cohort 1 i.n. dose) of YPP-401 starting at 18 h post-challenge and ~q6–12 h for a total of 4 doses until 42 hpi (Fig. 2). We showed a dose-dependent effect on survival after only four intermediate post-exposure doses of YPP-401 where the rate of survival increased to 88% (Fig. 2A) compared to 50% in the low dose i.n. group in cohort 1 (Fig. 1B). Although this increase in survival was not statistically significant via the log-rank test, no *Y. pestis* was detected in the spleen or liver of the surviving animals at the termination of the study (Table S3). In another study using 1 × 10^9^ PFU of a different phage cocktail, no efficacy with phage therapy was observed, suggesting a dose-dependent effectiveness for the treatment of pneumonic plague (27). Stringency of test parameters was increased further by assessing post-exposure efficacy at “late” times post-challenge (i.e., 42 hpi, after animals become symptomatic and a time where existing antibiotics have suboptimal efficacy; Fig. 3). “Late” post-exposure treatment with levofloxacin dosed daily for 10 days starting at 42 hpi provided a suboptimal survival rate of 50%. When YPP-401 was delivered starting at 42 hpi by i.p. or i.m. at 2× the cohort 1 dose, all animals succumbed; however, only one or two phage doses were able to be delivered prior to the time of death (Table S4).

It has been well documented that the timing of treatment for pneumonic plague is very important. Most literature suggests that if not treated within 24 h of symptom onset, pneumonic plague is almost uniformly fatal (10, 57, 58). As shown in this study, levofloxacin treatment initiated at 18 hpi is very effective at completely protecting rats from pneumonic plague. Byrne, et al. also have shown that ciprofloxacin, streptomycin, and ofloxacin treatments were 100% effective when administered 24 hpi in mice that were challenged with 100 LD_50_ of *Y. pestis* via the aerosol route. When treatment was delayed until 42 hpi, the efficacy of these three treatments dropped to ~60% (31). At a lower challenge dose of 12 LD_50_ using an i.n. route of infection in rats, instead of aerosol model, delaying levofloxacin treatment until 42 hpi was 100% protective but by further delaying the treatment to 48 hpi, protective efficacy of levofloxacin dropped to 44% (32). When cethromycin was used as the treatment, at 30 LD_50_ in the i.n. rat challenge model, delaying treatment past 24 hpi reduced survival from 100% at 24 hpi to 44% when treatment was started at 36 or 48 h post-infection (32). In the studies we presented here, rats were challenged with ~7,000 LD_50_ via the aerosol route; thus, it is not surprising that levofloxacin treatment only protected 50% of the rats and that the 1–2 doses of YPP-401 delivered starting at 42 hpi were suboptimal. Although each therapy (phage or antibiotic) alone was not effective at this late treatment timepoint, a few studies have evaluated combination therapies to enhance the efficacy at delayed times post-exposure. Combination therapy of phage with antibiotics could help extend the effective window for the treatment of pneumonic plague. Such studies form part of our future direction.

Overall, these proof-of-concept data demonstrate the promise of YPP-401 as a post-exposure treatment option to manage *Y. pestis* pneumonic infections. These studies are the first reported standalone use of effective phage therapy in an aerosol challenge model of pneumonic plague using rats. It provides promising data for future studies that could help usher phage therapy into the clinic for the treatment of pneumonic plague under the Animal Rule pathway for licensure (59).

## MATERIALS AND METHODS

### Bacterial strains

Twelve avirulent *Y. pestis* isolates (Table 1) were obtained through BEI Resources (Manassas, VA; supported by the National Institute of Health [NIH]/ NIAID). These 12 isolates are each lacking at least one of the four features essential for bacterial virulence (e.g., the pMT1, pPCP1, and pCD1 plasmids and the unstable pigmentation [*pgm*] locus, required for iron acquisition by bacteria from the host). Several are derivatives of known virulent strains (e.g., KIM, Yokohama). Fourteen *Yersinia* isolates, representing six non-*Y*. *pestis* species (Table 1), were provided by the University of Florida (Drs. Tamara Revazishvili and John Glenn Morris) and included four *Y. pseudotuberculosis* strains and two strains each of *Y. aldovae*, *Y. enterocolitica*, *Y. frederiksenii*, *Y. kristensenii*, and *Y. mollaretii*.

A representative panel of “core microbiome” species of the gastrointestinal (GI) tract was selected based on several studies comparing diverse healthy individuals (60–62). The main limitation when selecting the strains was the ability of the particular isolate to be cultured, as many *Bacteroidetes* and/or *Firmicutes* have never been cultured. Therefore, several species of common culturable bacterial genera from these phyla were identified and used. *E. coli* strains were selected, if they were isolated from healthy humans and not known to be associated with diseases, to represent members of the phylum *Proteobacteria*. These bacteria represent “healthy gut” *E. coli*. Common probiotic bacteria, such as *Bifidobacterium* spp. found in yogurts, are also associated with the healthy microbiome (phylum *Actinobacteria*) and were included as part of the panel. Many of the isolates from healthy human guts are also part of the BEI Resources Human Microbiome Project initiative, and the genomes of several have been sequenced by the Broad Institute. In all, 48 isolates representing 5 phyla and 13 genera were chosen to represent the core microbiome of the GI tract (Table S1).

#### Challenge strain

A fully virulent human pneumonic plague isolate, *Y. pestis* strain CO92 (biovar orientalis), was obtained from BEI Resources (63). *Y. pestis* CO92 was grown on tryptic soy broth (TSB) for all *in vitro* assays. Assay-specific growth conditions are described below. Cultures of *Y. pestis* CO92 were grown on plates at 28°C on the indicated medium (TSB, heart infusion broth [HIB], or sheep’s blood agar [SBA]). All studies involving *Y. pestis* CO92 were performed in Biosafety level (BSL)-3, CDC-approved Tier 1 select agent laboratories at the University of Texas Medical Branch (UTMB) in the Galveston National Laboratory (GNL; Galveston, TX). Prior to the initiation of the study, the standard *Y. pestis* phenotypes for strain CO92 were confirmed by growth on various differential media, and PCR on the well characterized *Y. pestis* virulence genes, *pla* (plasminogen activating protease), *caf*1 (capsular antigen F1), and *lcrV* (low calcium response V antigen), using genomic DNA (64, 65).

### YPP-401 bacteriophage cocktail

YPP-401 is an aqueous, buffered cocktail composed of approximately equal concentrations of four different and previously characterized monophages (24, 29) that were grown on *Y. pseudotuberculosis* and stored at 4°C protected from light until use. The YPP-401 formulation and monophage identities are proprietary to Intralytix, Inc., a company developing YPP-401 as a biothreat medical countermeasure. Using a proprietary software package, PhageSelector (developed by Intralytix), which facilitates the design of optimally effective phage preparations, we formulated the candidate YPP-401 for *Y. pestis*. The program uses various algorithms to analyze the database of bacterial strains in conjunction with phage sensitivity data to suggest which phages should be included in the preparations. For example, the program examines the data for phage potency variation (i.e., the total number of bacterial strains that each phage is capable of lysing) and analyzes the phage lysis efficiency (i.e., identifies the phages that are most diverse in their lytic activity against the strains listed in the database).

### *In vitro* phage susceptibility testing

Susceptibility of the challenge strain, *Y. pestis* CO92, to the YPP-401 cocktail was assessed using the same bacterial stock used for the *in vivo* challenge studies. *Y. pestis* CO92 was grown in TSB at 28°C overnight (O.N.; ~16 h) from a −80°C stock. The next morning, cultures were diluted 1:10 and grown for 1 h at 37°C (OD_600_ 0.3 ± 0.15). TSB containing 0.7% agar was autoclaved and kept in a 52°C water bath until use. Phage susceptibility assays were performed using the classical spot test (30) and liquid growth assays.

For the spot tests, the bacterial culture (100 µL) was combined with 3–5 mL of 0.7% top agar, overlaid on top of 1.5% bottom agar plates, and allowed to solidify. Serial dilutions (up to 10^−10^) of the phage cocktail were prepared. Each serial phage dilution was spotted (10 µL) individually onto the overlay plates. Plates were then incubated up to 48 h at the appropriate conditions for each bacterial species (listed in Table 1; Table S1) and then examined for zones of lysis. For the liquid-based phage susceptibility assay, 200 µL of an O.N. *Y. pestis* culture was combined with 800 µL of each serial phage cocktail dilution in duplicate and incubated at 28°C or 37°C, shaking at 180 RPM, for 16–24 h. Bacterial growth was measured using turbidity (OD_600_).

### *In vitro* antibiotic susceptibility testing

The antibiotic susceptibility testing was performed using the BD BBL Sensi-Disc Antimicrobial Susceptibility Test Discs (Becton, Dickinson and Co., Franklin Lakes, NJ) per the manufacturer’s instructions. Briefly, for each isolate, a suspension of colonies was prepared by selecting several colonies from the agar plate. The suspension was diluted, as required, to obtain turbidity equivalent to 0.5 McFarland turbidity standard. A sterile cotton swab was dipped into the inoculum and then streaked across the entire agar surface of the appropriate medium plates. The plates were allowed to dry, and then the appropriate discs were aseptically applied to the surface of the agar. The plates were incubated agar side up at the appropriate conditions for 16–24 h. The diameters of the zones of complete inhibition were measured to the nearest whole millimeter.

### Rat pneumonic plague challenge model

#### Animals

Brown Norway rats (*Rattus norvegicus*; 10–12 weeks old) were purchased from Charles River Laboratories (Houston, TX). Animals were acclimated for a minimum of 7 days in the Association for Assessment and Accreditation of Laboratory Animal Care International (AAALAC)-accredited, CDC-approved UTMB animal BSL-3 facility (ABSL-3) before the start of the experiment. Animals were provided food and water *ad libitum* and were maintained on a 12 h light dark cycle. Equal numbers of male (200–250 g) and female rats (100–150 g) of the same age were used in each experiment. All animal experiments were conducted under an approved Institutional Animal Care and Use Committee (IACUC) protocol.

#### Aerosol challenge

Rats were challenged as previously described with a lethal dose (28) using a fully automated computer-controlled rodent whole-body aerosol exposure system (Biaera AeroMP; Biaera Technologies, Hagerstown, MD). *Y. pestis* CO92 was prepared fresh prior to each challenge as previously described (28, 66). Briefly, the strain was streaked from a −80°C stock onto an SBA plate and incubated at 28°C for 48 h. The plate was then scraped, and the culture was resuspended in 4 mL of HIB. The culture was diluted 1:350 in 100 mL of HIB containing 0.2% xylose in a 500 mL HEPA-filter-capped flask, and incubated for 24 h at 30°C, shaking at 100 RPM. The culture was then washed twice in HIB and resuspended in 1/10th the original culture volume (i.e., 10 mL) of HIB. A further 1:10 dilution was performed, and Antifoam A Concentrate (Sigma-Aldrich, Saint Louis, MO) was added to a final concentration of 0.2%. The aerosol challenge preparation was then titrated on SBA plates after 10-fold serial dilutions to confirm bacterial concentration.

Separate aliquots were prepared for each aerosol exposure run since only eight rats could be accommodated in a single run. Aliquots of HIB (20 mL) supplemented with 4% glycerol and 0.2% Antifoam A were prepared for each run to quantify bacterial numbers in samples collected by an SKC BioSampler (SKC, Inc., Eighty Four, PA). The following aerosol parameters were used: an air inflow rate of 30 L/min, with 14 L/min to the nebulizer and 16 L/min to dilute the air; and an air outflow rate of 30 L/min, with 12.5 L/min to the bio-sampler and 17.5 L/min to the exhaust. The humidity was maintained at >60%, and the aerosol exposure time of *Y. pestis* to animals was 15 min using a 6-jet Collison nebulizer (CH Technologies [USA], Inc., Westwood, NJ). An Aerodynamic Particle Sizer (TSI, Inc., Shoreview, MN) was used to validate the aerosol delivery equipment, specifically the aerosolization efficiency (spray factor; SF), aerosol concentration derived from the samples (CSAMP), and the concentration of bacteria in the suspension pipetted into the nebulizer (CNEB). Due to practical limitations on number of animals that could be exposed to bacteria at one time, the challenge was divided into staggered consecutive runs. After bacterial exposure, rats were randomly distributed among treatment groups to account for any potential variability in presented dose (*D*_p_) between each run. The average *C*_neb_ (nebulizer concentration) for all aerosol runs was 3.42 × 10^9^ CFU/mL with a *D*_p_ to rats ranging from 9.56 × 10^6^ to 1.24 × 10^7^ CFUs for all runs. The *D*_p_ is calculated using the following formula: $D_{p}=\;C_{aero}\;x\;MV\;x\;T_{exp}$. *C*_aero_ is the aerosol concentration in the exposure chamber, MV is the minute volume calculated using Guyton’s formula based on the weight of the animal, and *T*_exp_ is the exposure time (67, 68). The spray factors for all aerosol runs ranged from 1.43 × 10^−6^ to 2.71 × 10^−6^.

#### Dosing regimen

As indicated, phage treatment was initiated at either 18 or 42 hpi. The levofloxacin antibiotic positive control (Akorn, Inc., Lake Forest, IL; NDC: 17478-107-20) was administered once daily at 50 mg/kg via the oral route for 10 days. The vehicle (i.e., placebo; 100 µL 1× PBS) control was administered i.p. starting at either 18 or 42 hpi, and as indicated. Oral administration of the phage preparation was performed as described (32) using disposable animal feeding needles (20G × 1.50 in.; Thermo Fisher Scientific, Waltham, MA). The volumes of each dose of YPP-401 for various routes were 500 µL oral; 500 µL i.p.; 500 µL i.m. at 250 µL per hind leg; and 100 µL i.n. at 50 µL per naris under anesthesia via inhalation of isofluorane. When treatment was initiated at 18 hpi, vehicle and YPP-401 were administered by the indicated route four times over 2 days (approximately every 6–12 h) at 18, 24, 30, and 42 hpi. When treatment was initiated at 42 hpi, vehicle and YPP-401 were intended to be administered by the indicated route four times over 2 days (approximately every 6–12 h) at 42, 48, 66, and 72 hpi. A number of doses actually administered when treatment was initiated at 42 hpi are indicated in Table S4.

#### Efficacy

Infected and treated rats were observed for morbidity and mortality twice per day for up to 14–15 days with three checks/day during 3–6 dpi or when animals exhibited increased clinical scores. Clinical outcome measures included rate of survival at 14 dpi, weight loss (measured every other day during 0–7 dpi and 2× per week thereafter), and clinical scores. Clinical scores were quantitative assessments using an independent variable score for appearance, activity level, respiration, and facial expression. The rodent clinical scoring rubric was approved by the UTMB IACUC.

### Quantification of bacterial load

Aerosol samples were collected in a SKC BioSampler and were 10-fold serially diluted and spotted on SBA plates to quantify bacterial numbers. To quantify bacteremia, the blood was collected at 42 hpi (and prior to initiation of the treatment for animals dosed at 42 hpi). Organs/tissues (i.e., lung, liver, heart, spleen) were collected from non-perfused moribund rats at the time of euthanasia or from surviving rats at 14–15 dpi (i.e., end of the study). Tissues were homogenized by placing the lung, spleen, or heart into 15 mL Closed Tissue Grinder Systems (Fisherbrand, Pittsburgh, PA) with 2 mL of Dulbecco’s PBS (DPBS). The liver was homogenized using 50 mL Closed Tissue Grinder System with 4 mL DPBS. Blood and tissue homogenates were 10-fold serially diluted in DPBS and then plated onto SBA plates to quantify bacterial load.

### Statistical methods

Statistical analysis was performed using GraphPad Prism version 10.0.0 for Windows (GraphPad Software, Boston, MA). Kaplan-Meier analysis with Logrank (Mantel-Cox) test was used for survival comparisons. As indicated, data are represented as the arithmetic mean with the standard error of the mean or the geometric mean with error bars showing geometric standard deviation. To compare the *Y. pestis* burden in blood at 42 hpi between treatment groups, a one-way analysis of variance (ANOVA) was conducted for each cohort, followed by Kruskal-Wallis test and then Dunn’s multiple comparisons test. To compare the terminal bacterial burden in each organ between treatment groups, a two-way ANOVA was conducted for each cohort, followed by Tukey’s post-hoc analysis for multiple comparisons for each organ.

## SUPPLEMENTAL MATERIAL

Supplemental figure and tables spectrum.00942-24-s0001.pdf Fig. S1; Tables S1 to S4.

ASM does not own the copyrights to Supplemental Material that may be linked to, or accessed through, an article. The authors have granted ASM a non-exclusive, world-wide license to publish the Supplemental Material files. Please contact the corresponding author directly for reuse.
