# Supplementary material for: A bacteriophage cocktail targeting Yersinia pestis provides strong post-exposure protection in a rat pneumonic plague model
Source: Microbiol Spectr. 2024 Sep 18;12(11):e00942-24. doi: 10.1128/spectrum.00942-24 (PMC11537065; doi:10.1128/spectrum.00942-24)
Supplement: Supplemental figure and tables — Fig. S1; Tables S1 to S4. [file spectrum.00942-24-s0001.pdf]

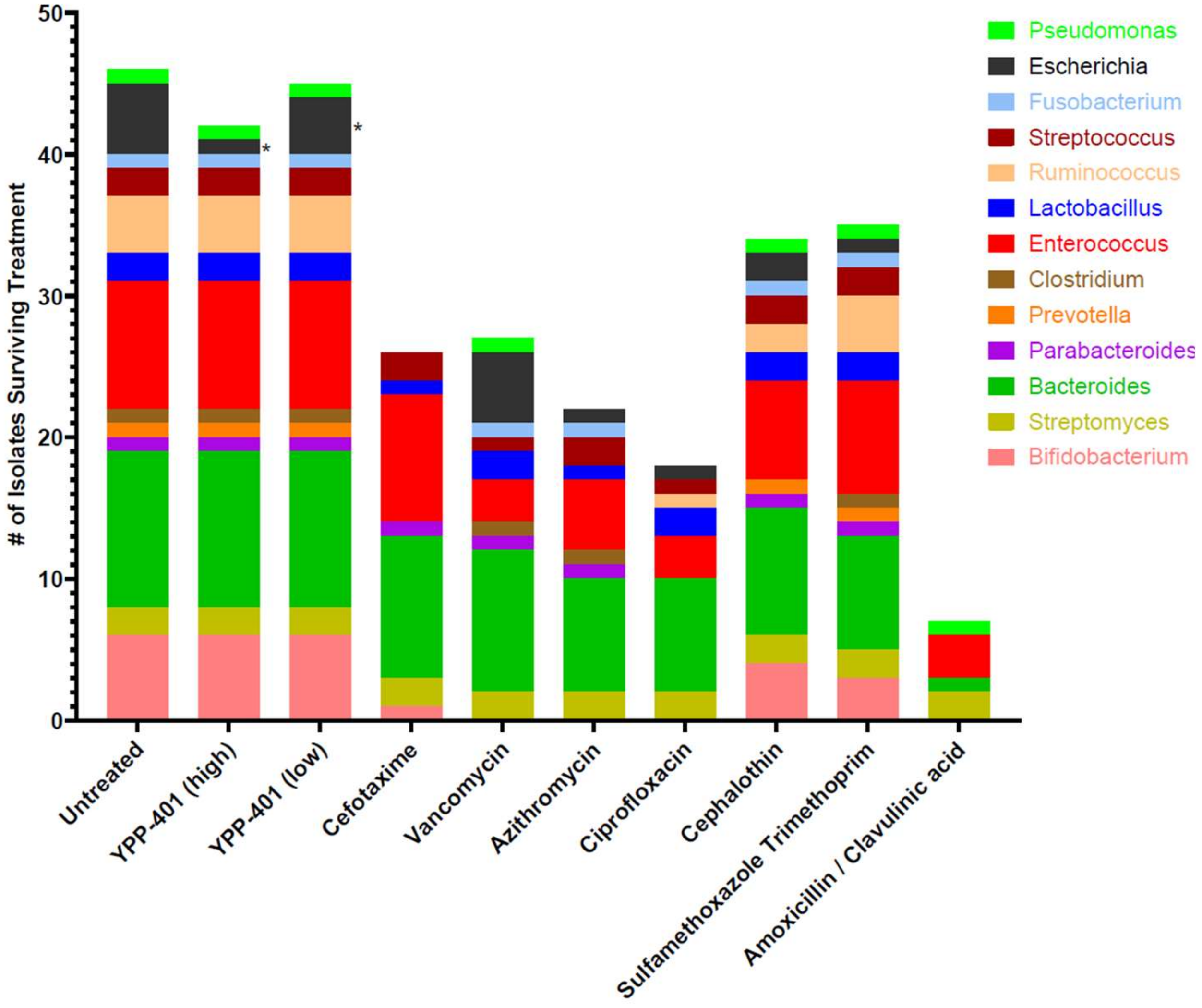

Figure S1. YPP-401 is Highly Specific for *Yersinia* spp. Compared to Antibiotics.

Specificity of YPP-401 for *Yersinia* spp. was assessed by testing susceptibility of 48 non-*Yersinia* bacterial strains representing 5 phyla. The phyla, genera and individual species are shown in **Table S1**. Each colored bar represents the number of strains within the indicated genera susceptible to the treatment on the x-axis. Susceptibility to YPP-401 at low (i.e., $2x10^4$ PFU/mL) or high (i.e., $1x10^9$ PFU/mL) concentration was assessed using spot and/or liquid growth assays. Susceptibility to the indicated antibiotic was performed using the BD BBL™ Sensi-Disc™ Antimicrobial Susceptibility Test Discs (Becton, Dickinson and Co., Franklin Lakes, NJ). Asterisks indicate only non-*Yersinia* genera tested (i.e., *Escherichia*; black bars) with susceptibility to YPP-401.

SUPPLEMENTARY TABLES

Table S1. Representative Non-Yersinia Host Microbiome Isolates for Specificity Testing

Phylum	Species	Source / Strain
Actinobacteria	*Bifidobacterium angulatum*	BEI HM-1189
	Bifidobacterium breve	BEI HM-856
	Bifidobacterium longum	ATCC 55813
	Bifidobacterium longum	ATCC 55814
	Bifidobacterium longum	ATCC BAA-999
	Bifidobacterium sp.	BEI HM-30
	Bifidobacterium/Lactobacillus sp.	ATCC 11146
	Streptomyces sp.	BEI HM-859
	Streptomyces sp.	BEI HM-789
Bacteroidetes	*Bacteroides caccae*	BEI HM-728
	Bacteroides dorei	BEI HM-717
	Bacteroides fragilis	ATCC 700786
	Bacteroides fragilis	BEI HM-710
	Bacteroides fragilis	BEI HM-20
	Bacteroides fragilis	BEI HM-714
	Bacteroides salyersiae	BEI HM-725
	Bacteroides sp.	BEI HM-18
	Bacteroides sp.	BEI HM-23
	Bacteroides sp.	BEI HM-22
	Bacteroides sp.	BEI HM-19
	Parabacteroides merdae	ATCC 43184
	Prevotella nigrescens	BEI HM-1053
	Prevotella oralis	BEI HM-1054
Firmicutes	*Clostridium innocuum*	BEI HM-173
	Enterococcus faecalis	ATCC BAA-2820
	Enterococcus faecalis	ATCC 51299
	Enterococcus faecalis	BEI HM-432
	Enterococcus faecalis	BEI HM-202
	Enterococcus faecalis	BEI NR-32002
	Enterococcus faecium	BEI HM-975
	Enterococcus faecium	BEI HM-952
	Enterococcus faecium	BEI HM-959
	Enterococcus faecium	BEI HM-204
	Lactobacillus brevis	ATCC 14869
	Ruminococcus gnavus	BEI HM-1056
	Ruminococcus lactaris	BEI HM-1057
	Ruminococcus sp.	BEI HM-79

Phylum	Species	Source / Strain
	Ruminococcus torques	ATCC 27756
	Ruminococcus torques	ATCC 35915
	Streptococcus parasanguinis	BEI HM-1060
	Streptococcus sanguinis	BEI HM-1061
Fusobacteria	*Fusobacterium ulcerans*	BEI HM-1194
Proteobacteria	[*]***Escherichia coli***	**BEI HM-366**
	[†]***Escherichia coli***	**BEI HM-365**
	[*]***Escherichia coli***	**BEI HM-347**
	[*]***Escherichia coli***	**BEI NR-17674**
	Escherichia coli	BEI NR-17676
	Pseudomonas sp.	BEI HM-214

[*]Non-*Yersinia* strains susceptible to YPP-401 at high (*i.e.,* $1x10^9$ PFU/mL) concentration only (**Figure S1**);

[†]Non-*Yersinia* strain susceptible to YPP-401 at high and low (*i.e.,* $2x10^4$ PFU/mL) concentrations (**Figure S1**).

Table S2. Clinical Measures and Bacterial Burden in Cohort 1 Rats Challenged at 18 hpi

Treatment	Route	ID	Sex	Clinical Score	TTD (Dpi)	Blood (CFU/mL)*	Lung (CFU/organ)‡	Liver (CFU/organ)‡	Spleen (CFU/organ)‡	Heart (CFU/organ)‡
Phage	p.o.	1	M	9	2.5	8.0×10^8	1.55×10^8	5.0×10^8	1.15×10^9	1.65×10^7
		7	M	9	2.5	5.0×10^8	5.5×10^5	2.9×10^6	1.1×10^3	2.0×10^3
		10	M	8	2.5	3.0×10^8	1.25×10^9	3.1×10^9	3.3×10^9	1.5×10^8
		16	M	2	6	2.2×10^3	7.0×10^8	1.3×10^8	1.2×10^8	5.0×10^6
		17	F	2	5	2.8×10^3	5.0×10^7	7.0×10^8	3.0×10^7	4.0×10^6
		22	F	1	2.5	1.1×10^8	3.5×10^5	3.7×10^6	3.0×10^2	5.0×10^4
		27	F	8	2.5	9.5×10^6	2.5×10^7	6.0×10^7	4.0×10^7	1.3×10^5
		32	F	8	2.5	1.75×10^8	1.65×10^5	2.8×10^6	2.4×10^6	4.0×10^3
Phage	i.p.	2	M	1	5	180	2.0×10^7	1.7×10^8	1.2×10^7	BDL
		8	M	0	N/A	330	ND	ND	ND	ND
		9	M	0	N/A	170	BDL	BDL	BDL	BDL
		15	M	0	N/A	BDL	ND	ND	ND	ND
		19	F	0	N/A	160	ND	ND	ND	ND
		21	F	0	N/A	10	ND	ND	ND	ND
		28	F	0	N/A	180	ND	ND	ND	ND
		30	F	0	N/A	BDL	BDL	BDL	BDL	BDL
Phage	i.n.	4	M	0	N/A	690	BDL	BDL	8.5×10^4	BDL
		5	M	0	N/A	80	ND	ND	ND	ND
		20	F	0	N/A	20	BDL	BDL	BDL	BDL
		29	F	0	N/A	1.53×10^5	ND	ND	ND	ND
		11	M	0	4	100	5.5×10^5	1.0×10^8	4.0×10^7	1.0×10^5
		13	M	2	6	2.0×10^3	1.6×10^6	1.4×10^7	6.5×10^7	5.8×10^3

Treatment	Route	ID	Sex	Clinical Score	TTD (Dpi)	Blood (CFU/mL)*	Lung	Liver	Spleen	Heart
							(CFU/organ)‡			
		24	F	0	9	BDL	3.5×10^{7}	2.5×10^{8}	7.0×10^{7}	9.5×10^{6}
		25	F	1	8	300	3.0×10^{7}	7.0×10^{7}	6.0×10^{7}	3.12×10^{4}
Levo	p.o.	3	M	0	N/A	10	BDL	BDL	BDL	BDL
		12	M	0	N/A	1.13×10^{3}	ND	ND	ND	ND
		23	F	0	N/A	60	BDL	BDL	BDL	BDL
		31	F	0	N/A	1.0×10^{3}	ND	ND	ND	ND
Vehicle	i.p.	6	M	8	2.5	4.5×10^{8}	1.3×10^{9}	6.0×10^{9}	6.5×10^{9}	1.85×10^{9}
		14	M	8	2.5	1.63×10^{8}	5.5×10^{8}	5.5×10^{9}	5.0×10^{9}	1.4×10^{8}
		18	F	3	3	1.75×10^{5}	3.05×10^{9}	2.0×10^{10}	3.75×10^{9}	1.2×10^{9}
		26	F	3	3	4.75×10^{7}	3.5×10^{9}	1.6×10^{10}	5.0×10^{9}	9.5×10^{8}

BDL, below detection limit (*i.e.,* 10 CFU/mL blood or 40 CFU/organ); ID, animal identification number; Levo, levofloxacin; N/A, not applicable because animal survived to end of study; ND, not determined; p.o., *per os*; TTD, time to death;
*Blood collected at 42 hpi, immediately before last phage dose;
‡Total organ burden (CFU/organ) was assessed at the terminal timepoint (time of death or euthanasia).

Table S3. Clinical Measures and Bacterial Burden in Cohort 2 Rats Challenged at 18 hpi

Treatment	Route	ID	Sex	Clinical Score¶	TTD (Dpi)	Blood (CFU/mL)*	Lung (CFU/organ)‡	Liver	Spleen	Heart
Phage	i.n.	1	M	0	N/A	BDL	BDL	BDL	BDL	BDL
		6	M	0	N/A	BDL	BDL	BDL	BDL	BDL
		11	M	0	N/A	BDL	2.1 x 10^6	BDL	BDL	6.5 x 10^4
		16	M	0	N/A	5.0 x 10^3	BDL	BDL	BDL	BDL
		22	F	0	N/A	BDL	BDL	BDL	BDL	BDL
		27	F	0	N/A	BDL	BDL	BDL	BDL	BDL
		32	F	0	N/A	5.0 x10^2	BDL	BDL	BDL	BDL
		17	F	0	6	BDL	4.5 x 10^2	4.9 x 10^7	3.1 x 10^8	4.0 x 10^3
Levo	p.o.	7	M	0	N/A	BDL	BDL	BDL	BDL	BDL
		18	F	0	N/A	BDL	BDL	BDL	BDL	BDL
Vehicle	i.p.	2	M	9	2.5	1.65 x 10^7	5.0 x 10^8	1.2 x 10^9	1.25 x 10^9	1.95 x 10^8
		21	F	7	3	3.0 x 10^6	2.0 x 10^6	8.0 x 10^3	2.0 x 10^4	4.5 x 10^3

BDL, below detection limit (i.e., 10 CFU/mL blood or 40 CFU/organ); ID, animal identification number; Levo, levofloxacin; N/A, not applicable because animal survived to end of study; ND, not determined; p.o., *per os*; TTD, time to death;
¶ Clinical score at time of death or euthanasia;
*Blood collected at 42 hpi, immediately before last phage dose;
‡Total organ burden (CFU/organ) was assessed at the terminal timepoint (time of death or euthanasia).

Table S4. Clinical Measures and Bacterial Burden in Cohort 3 Rats Challenged at 42 hpi

Treatment	Route	ID	Sex	# Doses†	Clinical Score¶	TTD (Dpi)	Blood (CFU/mL)*	Lung (CFU/organ)‡	Liver	Spleen	Heart
Phage	i.m.	3	M	2	7	3	7.5×10^5	1.6×10^8	1.9×10^9	9.5×10^8	1.05×10^8
		8	M	1	3	2.5	5.0×10^7	3.0×10^6	4.0×10^6	4.0×10^6	1.5×10^6
		10	M	2	7	3	2.5×10^4	5.5×10^6	1.7×10^7	1.5×10^6	1.0×10^6
		13	M	1	2	2.5	6.0×10^7	2.0×10^6	4.0×10^6	4.5×10^6	1.5×10^6
		19	F	2	7	3	1.75×10^5	3.0×10^8	5.0×10^9	7.5×10^8	1.1×10^8
		24	F	2	7	3	4.0×10^5	8.5×10^6	1.7×10^7	3.5×10^6	5.5×10^6
		25	F	2	7	3	1.0×10^5	2.5×10^6	2.2×10^8	4.5×10^7	2.0×10^6
		30	F	2	7	3	4.0×10^7	1.5×10^6	1.1×10^6	2.5×10^5	7.0×10^4
Phage	i.p.	4	M	2	7	3	7.0×10^5	1.9×10^8	1.4×10^9	6.0×10^8	1.8×10^8
		5	M	1	1	2.5	1.5×10^7	4.5×10^6	7.0×10^6	1.3×10^7	1.0×10^6
		9	M	1	0	2.5	5.0×10^7	3.5×10^6	8.0×10^6	7.5×10^6	1.5×10^6
		14	M	1	3	2.5	4.5×10^7	3.5×10^5	4.8×10^4	4.0×10^3	3.0×10^3
		20	F	1	9	2.5	1.0×10^5	7.5×10^4	1.0×10^3	4.0×10^3	4.5×10^2
		23	F	2	7	3	5.5×10^5	6.0×10^7	1.3×10^9	6.0×10^8	7.0×10^7
		28	F	2	7	3	1.4×10^6	6.0×10^7	1.6×10^9	1.25×10^7	4.0×10^7
		29	F	2	7	3	5.0×10^3	7.5×10^5	1.7×10^5	5.0×10^3	6.0×10^2
Vehicle	i.p.	12	M	1	9	2.5	1.3×10^7	4.0×10^9	7.0×10^9	7.0×10^9	5.0×10^8
		31	F	2	7	3	7.5×10^6	7.5×10^8	6.0×10^9	3.5×10^9	3.0×10^8
Levo	p.o.	15	M	10	0	N/A	6.5×10^4	BDL	BDL	BDL	BDL
		26	F	1	7	3	2.5×10^7	3.0×10^3	1.7×10^4	4.0×10^4	3.5×10^2

BDL, below detection limit (i.e., 10 CFU/mL blood or 40 CFU/organ); ID, animal identification number; Levo, levofloxacin; N/A, not applicable because animal survived to end of study; ND, not determined; p.o., *per os*; TTD, time to death;
† Total number of treatment doses each animal received prior to euthanasia or succumbing to death;
¶ Clinical score at time of death or euthanasia;

Treatment	Route	ID	Sex	# Doses†	Clinical Score¶	TTD (Dpi)	Blood (CFU/mL)*	Lung	Liver	Spleen	Heart
								(CFU/organ)‡			

* Blood collected at 42 hpi, immediately before first phage dose;
‡ Total organ burden (CFU/organ) was assessed at the terminal timepoint (time of death or euthanasia).
